# Positive and Negative Aspects of Public Insurance Coverage on ART With Respect to Multiple Gestation in Japan

**DOI:** 10.1002/rmb2.70078

**Published:** 2026-07-21

**Authors:** Aisaku Fukuda, Hiroshi Matsumoto, Isao Tsuji, Satoko Fujioka, Yoshiharu Morimoto

**Affiliations:** ^1^ IVF Osaka Clinic Higashi‐Osaka City Osaka Japan; ^2^ HORAC Grand Front Osaka Clinic Kita‐ku Osaka Japan

**Keywords:** assisted reproductive technology, embryo transfer, health insurance coverage, multiple embryo transfer, multiple pregnancy

## Abstract

**Purpose:**

This study examined whether the application of public insurance on assisted reproductive technology in Japan was associated with changes in the incidence of multiple blastocyst transfer (MBT) and subsequent multiple pregnancies (MP).

**Methods:**

A total of 2764 blastocyst transfer cycles performed at IVF Osaka Clinic from the pre‐insurance period (January 2020–March 2022) were retrospectively compared with 4,326 cycles from the insurance period (April 2022–February 2025). MP was defined as two or more gestational sacs.

**Results:**

Baseline analysis showed that patients' age after insurance coverage was significantly younger with fewer previous ET attempts than pre‐insurance. MBT rose from 5.8% pre‐insurance to 20.1% under insurance, and MP increased from 0.7% to 2.6%, simultaneously. Insurance coverage was independently associated with a higher likelihood of MBT, an association most pronounced in the older age cohort. MBT exhibited the highest risk profile for MP. Significant upward trends in MBT and MP were observed as patients approached their final insured cycles.

**Conclusions:**

Findings suggest a correlation between insured cycle limits and clinical practice toward prioritizing per‐cycle success. Although alleviating financial burden, the policy appears to be associated with increased MBT and MP, making it necessary to deliberate its impact on maternal and neonatal safety.

## 1 Introduction

In Japan, the first successful in vitro fertilization (IVF) was achieved at Tohoku University in 1983 [1]. Subsequently, the number of assisted reproductive technology (ART) cases in the country increased dramatically. This rapid expansion was enhanced by the development of ovarian stimulation protocols utilizing GnRH analogues and driven by the introduction of intracytoplasmic sperm injection (ICSI) in 1992 [2, 3].

Initially, due to relatively low pregnancy rates, Japan Society of Obstetrics and Gynecology (JSOG) practice committee allowed the number of embryos for transfer up to three. However, recognizing the subsequent rise in multiple pregnancy (MP), JSOG issued an important guideline in 2008 mandating that the number of embryos for transfer was restricted, in principle, to one [4]. This regulatory measure effectively reduced the MP rate resulting from IVF in Japan. Nonetheless, the recommendation included specific exceptions, allowing the transfer of two embryos for women aged 35 years or older or for patients who failed implantation from the first embryo transfer (ET) attempt.

A significant incidence of the Japanese governmental healthcare policy occurred in 2022. The new policy expanded the public health insurance coverage on infertility treatment including intrauterine insemination (IUI) and also IVF. The new insurance policy, however, was implemented with specific conditions on coverage, including limits on an applicable age of the female partner 42 or younger and also restrictions on the number of permitted ET cycles based on age: 6 times for 39 or younger and 3 times for 40–42 [5].

Much less economic burden on the patients' finance provided by the commencement of insurance coverage on ART led to an abrupt increase in the number of patients receiving ART [6]. Furthermore, specific demographic changes appeared, including two distinct peaks in the number of treatment cycles by female patient age, with the first peak at age 42, just below the insurance coverage cutoff of 43 years, and then the second peak at age 39, just before 40 years old when the number of limited ET cycles drops from six to three [7].

At the same time, concerns have emerged that the limitation on the number of ET cycles may have encouraged the increase in multiple embryo transfers (METs) under insurance coverage, as patients and clinicians strive to maximize the likelihood of pregnancy while preserving their limited number of permitted transfer cycles. A potential negative consequence of insurance coverage on ART is therefore the resurgence of multiple gestations driven by such practice attitude based on insurance policy.

The present study was conducted to clarify whether the incidence of MET, focusing specifically on blastocyst transfer, increased after the expansion of governmental insurance coverage on ART treatment in 2022. Therefore, we analyzed the underlying changes in the individual characteristics of blastocyst transfer cases with their background information subsequent to the implementation of insurance coverage on ART. Furthermore, by verifying the changes in the number of MET cycles and resulting MP, this research aims to demonstrate the aftermath of the ART practice under the new insurance policy and prospect future challenges.

## 2 Materials and Methods

### 2.1 Study Design and Population

**FIGURE 1:**
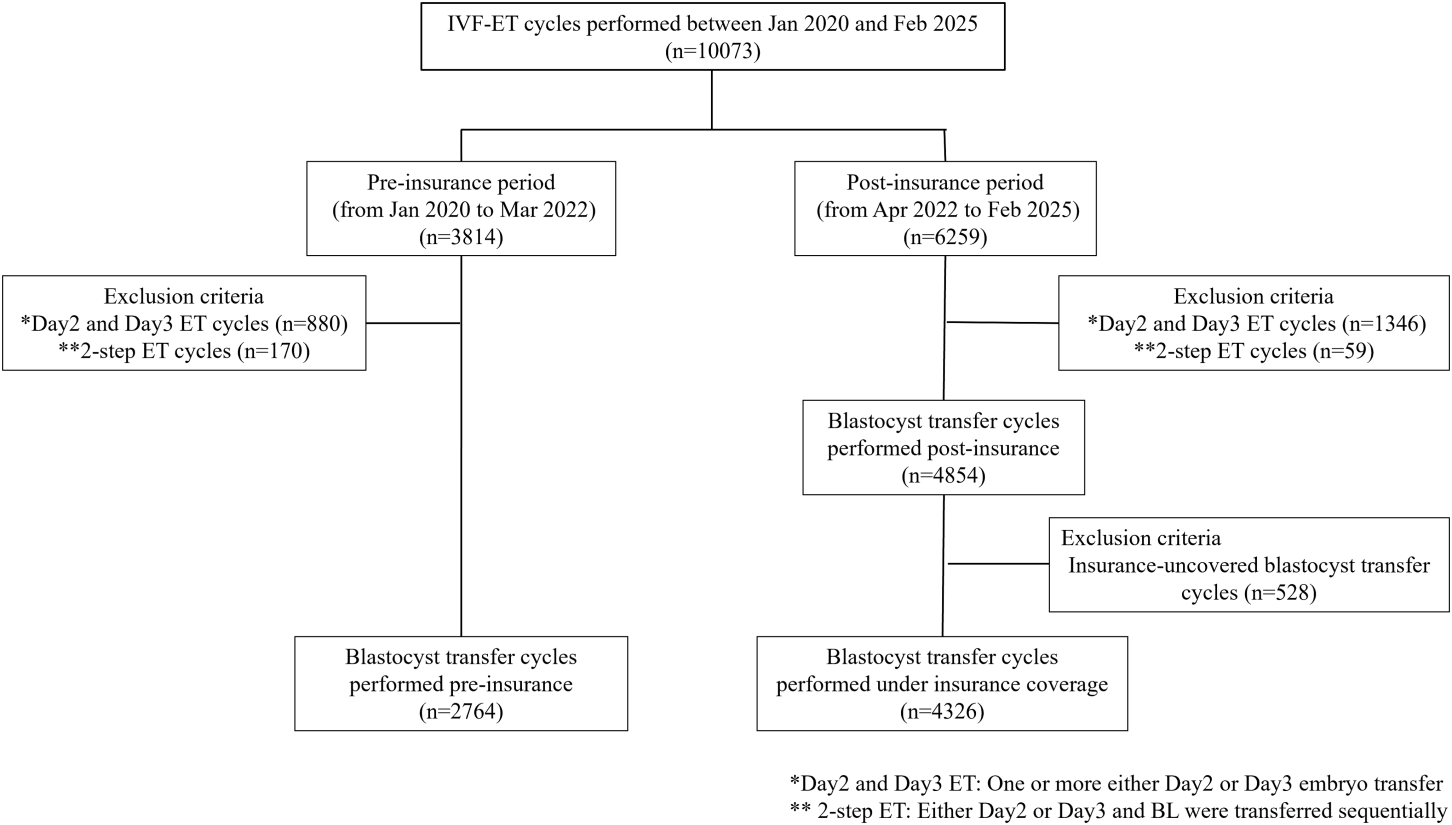
Flowchart shows the inclusion and exclusion criteria.

This retrospective cohort study was designed to investigate a large cohort of blastocyst transfer cycles performed at IVF Osaka Clinic. Between January 2020 and February 2025, a total of 10 073 ET cycles were performed. These were divided into two groups, specifically pre‐insurance period (January 2020 to March 2022; *n* = 3814) and post‐insurance period (April 2022 to February 2025; *n* = 6259). Throughout the study period, our clinical policy remained consistent, with single blastocyst transfer positioned as the first‐line treatment to minimize MP. Cleavage‐stage ET (Day 2 or Day 3) and 2‐step ET, defined as the sequential transfer of both a cleavage‐stage embryo and a blastocyst, were utilized only as alternative strategies for the patients predicted to have difficulty reaching the blastocyst stage. When considering the option of transferring multiple embryos, we proceeded with caution in accordance with the JSOG guideline, which allows double embryo transfer for women aged 35 years or older or for those who have not achieved pregnancy after two or more prior transfers. Importantly, these treatment policies were not changed after the introduction of health insurance coverage. To establish an analytic cohort of blastocyst transfer, we excluded cycles involving cleavage‐stage ET (*n* = 880 pre‐insurance; *n* = 1346 post‐insurance) and 2‐step ET (*n* = 170 pre‐insurance; *n* = 59 post‐insurance). Within the post‐insurance group, 528 blastocyst transfer cycles performed without insurance coverage were also excluded. The final analysis included 7090 blastocyst transfer cycles (2764 pre‐insurance and 4326 post‐insurance), as illustrated in the flow diagram (Figure 1).

### 2.2 Oocyte Retrieval and IVF

Ovarian stimulation was performed depending on each patient's medical history, in accordance with previously described standard protocols [8]. Ovulation was triggered with recombinant human chorionic gonadotropin (Ovidrel, Merck Serono, Darmstadt, Germany) when at least one dominant follicle reached 18 mm in diameter. Transvaginal oocyte retrieval was carried out 36 h after the trigger. The collected cumulus–oocyte complexes were cultured in fertilization medium (G‐IVF, Vitrolife, Gothenburg, Sweden). Insemination was performed by either conventional IVF or intracytoplasmic sperm injection depending on semen quality. Fertilization was confirmed by the presence of two distinct pronuclei with second polar body 16–20 h after insemination and subsequently cultured in the medium (Continuous Single Culture, FUJIFILM Irvine Scientific, Santa Ana, CA, USA) for further development until day 5 or 6. The grading of the blastocyst was evaluated with degrees of blastocyst expansion, inner cell mass quality, and trophectoderm quality using Gardner's classification system [9]. Blastocysts exhibiting an expansion stage greater than 2, along with inner cell mass and trophectoderm grades of A or B, respectively, were classified as high‐quality.

### 2.3 Blastocyst Cryopreservation and Thawing

In case of high risk of ovarian hyperstimulation syndrome, entire acquired blastocysts were cryopreserved using the vitrification method with a vitrification kit (Cryotop Safety Kit, Kitazato Corporation, Shizuoka, Japan). These blastocysts were subsequently warmed and transferred in a later cycle.

### 2.4 Blastocyst Transfer

Blastocyst transfer was performed on either fresh or frozen–thawed cycles. In fresh cycles, blastocysts were transferred 5 days after oocyte retrieval in patients with an endometrial thickness over 8 mm. Daily progesterone doses of 90 mg (Crinone, Merck Serono, Darmstadt, Germany) were administered until 9 weeks of gestation. In frozen–thawed cycles, the endometrium was prepared in two ways. In hormone replacement cycles, incremental doses of oral estradiol (Estradiol Tablets, Fuji Pharma Co. Ltd., Tokyo, Japan) ranging from 1 to 4 mg over 2 weeks were administered. After confirming an endometrial thickness over 8 mm by transvaginal ultrasound, 600 mg of progesterone (Utrogestan, Fuji Pharma Co. Ltd., Tokyo, Japan) was started to be administered from the next day for 6 days. Frozen–thawed blastocysts were transferred on the 6th day of progesterone administration. After ET, daily doses of 4 mg of estradiol and 600 mg of progesterone were administered until 9 weeks of gestation. In natural ovulation cycles, follicular development was followed by transvaginal ultrasound and once follicles reached 18 mm in diameter, recombinant human chorionic gonadotropin was administered and also LH was measured to set the day of blastocyst transfer. When LH was < 15 IU/L, ovulation was supposed to occur 2 days later. On the other hand, when LH was 15 IU/L or higher, ovulation was supposed to occur the next day. In both cases, 600 mg of progesterone was administered for 5 days from the estimated ovulation date. Frozen–thawed blastocysts were transferred on the fifth day of progesterone administration. Daily doses of 600 mg of progesterone were maintained until 9 weeks of gestation. Clinical pregnancy was defined when a gestational sac was confirmed by transvaginal ultrasonography beyond 5 weeks of gestation. The presence of two or more gestational sacs was defined as the criterion for diagnosing MP.

### 2.5 Statistical Analysis

Baseline characteristics of the patients and blastocyst transfer cycles were compared using the Mann–Whitney *U* test for continuous variables. The Pearson's chi‐square test was used to compare categorical variables. We performed univariable and multivariable analyses to identify factors associated with the decision to transfer multiple blastocysts. Generalized estimating equation (GEE) models with logit functions were used to evaluate the outcomes, accounting for multiple cycles per patient. The model incorporated covariates including maternal age, male partner's age, history of previous pregnancy, history of miscarriage, history of childbirth, the number of previous ETs received, tubal factor, thyroid dysfunction, the type of embryos transferred (fresh or frozen–thawed), transfer of at least one high‐quality blastocyst, and health insurance coverage with the pre‐insurance period serving as the reference.

Furthermore, the association between clinical factors and incidence of MP was assessed through two different GEE models. The initial model was performed without adjustment for the practice of multiple blastocyst transfer (MBT). The covariates included were patient age, male partner's age, history of previous pregnancy, history of miscarriage, history of childbirth, number of ETs received, tubal factor, thyroid dysfunction, type of embryos transferred, endometrial thickness, transfer of at least one high‐quality blastocyst, and health insurance coverage. The subsequent model included adjustment for MBT to assess its confounding role. The same set of covariates from the initial model was analyzed, with MBT included as an additional covariate.

To evaluate the structural impact of age‐dependent limitations within the Japanese health insurance system, we performed age‐stratified subgroup analyses. Patients were categorized into three groups based on age at ET (< 35 years, 35–39 years, and 40–43 years). For each group, the association between health insurance coverage and the decision of MBT was analyzed. The models were adjusted for maternal age, male partner's age, history of previous pregnancy, history of miscarriage, history of childbirth, the number of previous ETs received, tubal factor, thyroid dysfunction, the type of embryos transferred, transfer of at least one high‐quality blastocyst, and health insurance coverage. Furthermore, a statistical interaction between age category and health insurance coverage was assessed to determine whether the impact of the insurance policy on the decision making of MBT significantly varied across the age groups.

Moreover, the impact of insurance policy restrictions on clinical decision‐making was investigated. A stratified analysis focusing exclusively on blastocyst transfer cycles under the health insurance coverage period was performed. Patients were categorized into two groups based on their age at the time of oocyte retrieval (< 40 and 40–42 years), reflecting different cycle limits (up to six and three ET cycles, respectively). The rates of MBT and MP according to the number of previous insured ET cycles within each age category were evaluated. The Cochran‐Armitage trend test was employed to determine the statistical significance of trends across the increasing number of ET cycles.

A *p* < 0.05 was considered statistically significant. All statistical analyses were performed using EZR software, version 1.68 [10].

## 3 Results

### 3.1 Study Population and Characteristics

The present study investigated 2764 blastocyst transfer cycles conducted in the pre‐insurance period and 4,326 cycles under health insurance coverage. The baseline characteristics of the patients and blastocyst transfer cycles for both groups were summarized in Table 1. Patients who underwent blastocyst transfer under health insurance coverage were significantly younger compared to those in the pre‐insurance period. Similarly, their male partners were also significantly younger. There was no significant difference in body mass index (BMI) between the two groups. The cycles conducted under health insurance coverage showed a significantly higher proportion of the patients with histories of previous pregnancy, miscarriage, and childbirth. The average number of previous ETs in the pre‐insurance group was significantly higher compared to the insurance group. Regarding the etiology of infertility, the proportion of tubal factor infertility was significantly higher in those who underwent blastocyst transfer under health insurance coverage, whereas the prevalence of thyroid dysfunction was significantly lower. Other etiologies such as male factor, myoma, endometrioma, hyperprolactinemia, and adenomyosis showed no significant differences between the two groups. The type of blastocysts transferred also differed, with a significantly lower percentage of frozen–thawed blastocysts used in the cycles under health insurance coverage. Endometrial thickness at transfer was significantly greater in the cycles under health insurance coverage. The insurance coverage group had a significantly higher rate of transferring at least one high‐quality blastocyst. A major difference was also recognized in transfer strategy, particularly in the number of blastocysts transferred. The rate of MBT increased from 5.8% in the pre‐insurance period to 20.1% under health insurance coverage. Consistent with these changes, the clinical pregnancy rate was significantly higher in the cycles under health insurance coverage compared to the pre‐insurance period (46.4% vs. 41.9%). This was accompanied by an increase in the MP rate, which rose to 2.6% under health insurance coverage from 0.7% in the pre‐insurance period.

**TABLE 1:** Baseline characteristics of patients and blastocyst transfer cycles.

Characteristic	Pre‐insurance period (*n* = 2764)	Under health insurance coverage (*n* = 4326)	*p*
Age (y)	36.5 ± 4.2	35.8 ± 4.2	< 0.001
Male partner's age (y)	39.0 ± 5.7	38.0 ± 5.9	< 0.001
BMI (kg/m^2^)	21.4 ± 3.3	21.6 ± 3.7	0.336
History of previous pregnancy	1512 (54.7)	2548 (58.9)	0.001
History of miscarriage	747 (27.0)	1303 (30.1)	0.005
History of childbirth	984 (35.6)	1739 (40.2)	< 0.001
Number of ETs received	1.8 ± 2.0	1.7 ± 2.0	0.003
Etiology of infertility			
Male	1829 (66.2)	2808 (64.9)	0.282
Myoma	549 (19.9)	798 (18.4)	0.145
Tubal	462 (16.7)	852 (19.7)	0.002
Endometrioma	387 (14.0)	590 (13.6)	0.672
Thyroid dysfunction	568 (20.5)	687 (15.9)	< 0.001
Hyperprolactinemia	206 (7.5)	278 (6.4)	0.101
Adenomyosis	93 (3.4)	160 (3.7)	0.471
Type of blastocysts transferred (Frozen–thawed)	2362 (85.5)	3462 (80.0)	< 0.001
Endometrial thickness at transfer (mm)	11.1 ± 1.9	11.4 ± 2.2	0.003
Transfer of at least one high‐quality blastocyst	2182 (78.9)	3740 (86.5)	< 0.001
Multiple blastocyst transfer	161 (5.8)	871 (20.1)	< 0.001
Clinical pregnancy	1159 (41.9)	2007 (46.4)	< 0.001
Multiple pregnancy	18 (0.7)	114 (2.6)	< 0.001

*Note:* Data presented as mean ± SD or *n* (%).

Abbreviation: BMI, body mass index.

### 3.2 Factors Associated With the Decision of Multiple Blastocyst Transfer

The results of the univariable and multivariable analyses regarding the factors associated with the decision‐making to perform MBT are summarized in Table 2. After adjusting for all explanatory variables, including female patient age, male partner's age, history of previous pregnancy, miscarriage, and childbirth, the number of ETs received, tubal factor, thyroid dysfunction, embryo type, quality, and insurance status, several variables demonstrated significant associations with the decision‐making. Female patient age was significantly associated with a higher likelihood of choosing MBT (adjusted odds ratio [aOR] 1.08; 95% confidence interval [CI], 1.06–1.11; *p* < 0.001). Regarding clinical history, while a history of miscarriage showed a significant association in the univariable analysis (crude odds ratio [cOR] 1.73; 95% CI, 1.51–1.99; *p* < 0.001), this association was not significant after adjustment (aOR 0.999; 95% CI, 0.788–1.26; *p* = 0.995). Conversely, a history of childbirth was associated with a significantly reduced decision for MBT (aOR 0.338; 95% CI, 0.267–0.428; *p* < 0.001). The number of previous ETs received by the patient was positively correlated with the decision of MBT (aOR 1.49; 95% CI, 1.42–1.56; *p* < 0.001). The introduction of health insurance coverage demonstrated a strong association, with the likelihood of MBT being higher under insurance coverage compared to the pre‐insurance period (aOR 6.59; 95% CI, 5.19–8.36; *p* < 0.001). Other factors, including male partner's age, tubal factor, thyroid dysfunction, the type of embryos transferred, and the transfer of at least one high‐quality blastocyst, did not reach statistical significance in the multivariable model.

**TABLE 2:** Factors associated with the decision of multiple blastocyst transfer.

Explanatory variable	cOR (95% CI)	*p* for cOR	aOR (95% CI)^a^	*p* for aOR
Age	1.10 (1.08–1.12)	< 0.001	1.08 (1.06–1.11)	< 0.001
Male partner's age	1.04 (1.03–1.05)	< 0.001	1.00 (0.990–1.02)	0.408
History of previous pregnancy				
Absent	Reference		Reference	
Present	1.14 (0.998–1.30)	0.051	1.16 (0.899–1.51)	0.244
History of miscarriage				
Absent	Reference		Reference	
Present	1.73 (1.51–1.99)	< 0.001	0.999 (0.788–1.26)	0.995
History of childbirth				
Absent	Reference		Reference	
Present	0.718 (0.624–0.827)	< 0.001	0.338 (0.267–0.428)	< 0.001
Number of ETs received	1.45 (1.40–1.50)	< 0.001	1.49 (1.42–1.56)	< 0.001
Tubal factor				
Absent	Reference		Reference	
Present	1.12 (0.955–1.33)	0.154	0.897 (0.734–1.09)	0.289
Thyroid dysfunction				
Absent	Reference		Reference	
Present	0.968 (0.812–1.15)	0.721	0.826 (0.665–1.02)	0.085
Type of embryos transferred				
Fresh embryo	Reference		Reference	
Frozen–thawed embryo	0.912 (0.772–1.07)	0.285	0.848 (0.712–1.00)	0.064
Transfer of at least one high‐quality blastocyst				
No	Reference		Reference	
Yes	0.820 (0.692–0.973)	0.022	0.956 (0.775–1.17)	0.675
Health insurance coverage				
Pre‐Insurance	Reference		Reference	
Under health insurance coverage	4.06 (3.41–4.84)	< 0.001	6.59 (5.19–8.36)	< 0.001

Abbreviations: aOR; adjusted odds ratio; CI; confidence interval; cOR; crude odds ratio.

^a^ The model was adjusted for age, male partner's age, history of previous pregnancy, history of miscarriage, history of childbirth, number of ETs received, tubal factor, thyroid dysfunction, type of embryos transferred, transfer of at least one high‐quality blastocyst, and health insurance coverage.

### 3.3 Factors Associated With Multiple Pregnancy

The results of the univariable and multivariable analyses identifying factors associated with MP are summarized in Table 3. In the univariable analyses, several clinical factors were significantly associated with an increased or decreased risk of MP. A history of previous pregnancy (cOR 0.697; 95% CI, 0.494–0.984; *p* = 0.040) and childbirth (cOR 0.573; 95% CI, 0.388–0.846; *p* = 0.005) were both associated with lower odds. Conversely, the number of ETs received (cOR 1.19; 95% CI, 1.14–1.25; *p* < 0.001), the transfer of at least one high‐quality blastocyst (cOR 4.20; 95% CI, 1.85–9.57; *p* < 0.001), MBT (cOR 47.8; 95% CI, 28.2–81.0; *p* < 0.001), and health insurance coverage (cOR 4.12; 95% CI, 2.50–6.80; *p* < 0.001) were significantly associated with higher odds of MP. In the initial model of GEE, with adjustment for clinical variables excluding MBT, history of childbirth remained significantly negatively associated with the incidence of MP (aOR 0.462; 95% CI, 0.265–0.805; *p* = 0.006). Factors associated with increased odds of MP were the number of ETs received (aOR 1.30; 95% CI, 1.23–1.38; *p* < 0.001), transfer of at least one high‐quality blastocyst (aOR 4.88; 95% CI, 1.93–12.3; *p* < 0.001), and health insurance coverage (aOR 4.15; 95% CI, 2.48–6.94; *p* < 0.001). In the subsequent model, which included adjustment for MBT, the associations for history of childbirth, number of ETs received, and health insurance coverage were not significant (*p* > 0.05). Instead, maternal age emerged as a significant factor (aOR 0.902; 95% CI, 0.848–0.959; *p* = 0.001). The transfer of at least one high‐quality blastocyst maintained an association (aOR 4.94; 95% CI, 2.13–11.4; *p* < 0.001). MBT exhibited the highest risk profile for MP (aOR 61.2; 95% CI, 33.8–110.8; *p* < 0.001). Factors such as male partner's age, history of miscarriage, tubal factor, thyroid dysfunction, type of embryos transferred, and endometrial thickness did not show significant associations in any of the models.

**TABLE 3:** Association between clinical factors and multiple pregnancy: With and without adjustment for multiple blastocyst transfer.

Explanatory variable	cOR (95% CI)	*p* for cOR	Without adjustment for multiple blastocyst transfer^a^		With adjustment for multiple blastocyst transfer^b^	
			aOR (95% CI)	*p* for aOR	aOR (95% CI)	*p* for aOR
Age (y)	0.966 (0.928–1.00)	0.089	0.971 (0.912–1.03)	0.365	0.902 (0.848–0.959)	0.001
Male partner's age (y)	0.981 (0.949–1.01)	0.255	0.998 (0.952–1.04)	0.944	1.00 (0.956–1.04)	0.953
History of previous pregnancy						
Absent	Reference		Reference			
Present	0.697 (0.494–0.984)	0.040	0.956 (0.515–1.77)	0.887	0.830 (0.442–1.55)	0.562
History of miscarriage						
Absent	Reference		Reference			
Present	0.993 (0.679–1.45)	0.974	0.768 (0.440–1.33)	0.351	0.716 (0.410–1.25)	0.241
History of childbirth						
Absent	Reference		Reference			
Present	0.573 (0.388–0.846)	0.005	0.462 (0.265–0.805)	0.006	0.997 (0.575–1.72)	0.991
Number of ETs received	1.19 (1.14–1.25)	< 0.001	1.30 (1.23–1.38)	< 0.001	0.995 (0.910–1.08)	0.913
Tubal factor						
Absent	Reference		Reference			
Present	0.976 (0.624–1.52)	0.916	0.803 (0.497–1.29)	0.370	0.855 (0.530–1.37)	0.521
Thyroid dysfunction						
Absent	Reference		Reference			
Present	0.778 (0.476–1.27)	0.316	0.808 (0.467–1.39)	0.447	0.967 (0.570–1.64)	0.902
Type of embryos transferred						
Fresh embryo	Reference		Reference		Reference	
Frozen–thawed embryo	1.08 (0.685–1.72)	0.718	1.01 (0.631–1.62)	0.956	1.04 (0.631–1.71)	0.875
Endometrial thickness	0.988 (0.924–1.05)	0.735	1.01 (0.947–1.08)	0.693	1.01 (0.929–1.09)	0.813
Transfer of at least one high‐quality blastocyst						
No	Reference		Reference		Reference	
Yes	4.20 (1.85–9.57)	< 0.001	4.88 (1.93–12.3)	< 0.001	4.94 (2.13–11.4)	< 0.001
Multiple Blastocyst Transfer						
No	Reference		NA	NA	Reference	
Yes	47.8 (28.2–81.0)	< 0.001	NA	NA	61.2 (33.8–110.8)	< 0.001
Health insurance						
Pre‐Insurance	Reference		Reference		Reference	
Under health insurance coverage	4.12 (2.50–6.80)	< 0.001	4.15 (2.48–6.94)	< 0.001	1.46 (0.847–2.51)	0.172

Abbreviations: aOR; adjusted odds ratio; CI; confidence interval; cOR; crude odds ratio.

^a^ The model was adjusted for age, male partner's age, history of previous pregnancy, history of miscarriage, history of childbirth, number of ETs received, tubal factor, thyroid dysfunction, type of embryos transferred, endometrial thickness, transfer of at least one high‐quality blastocyst, and health insurance coverage.

^b^ The model was adjusted for age, male partner's age, history of previous pregnancy, history of miscarriage, history of childbirth, number of ETs received, tubal factor, thyroid dysfunction, type of embryos transferred, endometrial thickness, transfer of at least one high‐quality blastocyst, multiple blastocyst transfer, and health insurance coverage.

### 3.4 Age‐Stratified Analysis of the Association Between Health Insurance Coverage and the Decision of Multiple Blastocyst Transfer

Age‐stratified subgroup analyses showed the incidence of MBT before and under health insurance coverage across different age categories (Table 4). In the group of age < 35 years, the incidence of MBT was 5.5% (48/879) before insurance coverage and 12.0% (188/1566) under coverage, with an aOR of 3.39 (95% CI: 2.15–5.35). In the group of 35–39 years, the MBT rate was 5.5% (64/1159) before insurance coverage and 19.1% (340/1780) under coverage, resulting in an aOR of 5.16 (95% CI: 3.54–7.51). In the group of 40–43 years, the MBT rate was 7.0% (46/654) before insurance coverage and 35.0% (343/980) under coverage, with an aOR of 10.4 (95% CI: 6.85–15.9). The interaction between age categories and health insurance status was statistically significant (*p* < 0.001).

**TABLE 4:** Association between health insurance coverage and the decision to perform multiple blastocyst transfer, stratified by age group.

Age category	Health insurance	Multiple blastocyst transfer	cOR (95% CI)	aOR (95% CI)^a^	*p* for interaction
< 35 years	Pre‐insurance	(48/879, 5.5%)	Reference	Reference	< 0.001
	Under health insurance coverage	(188/1566, 12.0%)	2.49 (1.73–3.59)	3.39 (2.15–5.35)	
35–39 years	Pre‐insurance	(64/1159, 5.5%)	Reference	Reference	
	Under health insurance coverage	(340/1780, 19.1%)	3.61 (2.65–4.92)	5.16 (3.54–7.51)	
40–43 years	Pre‐insurance	(46/654, 7.0%)	Reference	Reference	
	Under health insurance coverage	(343/980, 35.0%)	7.06 (4.92–10.1)	10.4 (6.85–15.9)	

Abbreviations: aOR; adjusted odds ratio; CI; confidence interval; cOR; crude odds ratio.

^a^ The model was adjusted for age, male partner's age, history of previous pregnancy, history of miscarriage, history of childbirth, number of ETs received, tubal factor, thyroid dysfunction, type of embryos transferred, transfer of at least one high‐quality blastocyst, and health insurance coverage.

### 3.5 Multiple Blastocyst Transfer and Multiple Pregnancy Rates by Previous Insured Cycles

During the health insurance coverage period, both MBT and MP rates demonstrated a significant upward trend in proportion to the number of previous insured ET cycles increased (Table 5). Among patients under 40 years of age, the MBT rate was 4.4% in the first insured ET cycle (0 previous cycles) and increased to 74.6% by the sixth, the last insured ET cycle (5 previous cycles). Correspondingly, the MP rate in this age group rose from 1.1% in the first insured ET cycle to 11.1% in the sixth insured cycle. Both trends were statistically significant (*p* < 0.001). Similarly, for patients aged 40–42 years, the MBT rate increased from 15.0% in the first insured ET cycle (0 previous cycles) to 72.6% in the third, the last insured ET cycle (2 previous cycles). The MP rate also showed an increase from 0.5% to 6.2% over the same period. These increases were also statistically significant (*p* < 0.001).

**TABLE 5:** Multiple blastocyst transfer and multiple pregnancy rates stratified by the number of previous insured embryo transfers during the health insurance coverage period.

Insurance age category at the time of oocyte retrieval	Number of previous insured ET cycles	Total cycles	Multiple blastocyst transfer, *n* (%)	Multiple pregnancy, *n* (%)
< 40 years	0 (First cycle)	1689	75 (4.4)	18 (1.1)
	1	930	99 (10.6)	17 (1.8)
	2	510	188 (36.9)	18 (3.5)
	3	282	148 (52.5)	27 (9.6)
	4	132	80 (60.6)	11 (8.3)
	5	63	47 (74.6)	7 (11.1)
	*p* for trend		< 0.001	< 0.001
40–42 years	0 (First cycle)	387	58 (15.0)	2 (0.5)
	1	220	94 (42.7)	7 (3.2)
	2	113	82 (72.6)	7 (6.2)
	*p* for trend		< 0.001	< 0.001

## 4 Discussion

The present investigation provides a comprehensive evaluation of the clinical shifts in ART practice following the expansion of public health insurance coverage on infertility treatment in Japan in 2022. Our findings revealed a significant increase in the incidence of MBT, rising from 5.8% in the pre‐insurance period to 20.1% under the health insurance coverage, accompanied by a fourfold elevation in the MP rate (0.7%–2.6%). Although the revised health insurance policy has successfully reduced the economic burden on infertile patients, these observations suggest a shift in clinical practice that may be concurrently associated with an increase of MP.

An important finding at the time of insurance policy change was the significant demographic shift of the patient toward a younger population (both female and male sides). From an epidemiological perspective, this likely reflects the benefit of insurance coverage in lowering the financial burden for ART, encouraging younger couples to seek treatment earlier in their reproductive timeline rather than deferring care due to cost. This demographic transition is consistent with our observation of a higher rate of high‐quality blastocyst transfers in the insurance group. Younger age is a well‐established predictor of superior oocyte quality and embryonic developmental competence, leading to a higher yield of morphologically optimal blastocysts [11]. Furthermore, the insurance group exhibited a distinct clinical and etiological profile. A significantly higher prevalence of tubal factor infertility and a lower prevalence of thyroid dysfunction in this cohort were observed. Thyroid abnormalities are known to increase with age, and the younger demographic profile in the insurance group could contribute to this difference [12, 13]. The higher proportion of tubal factor infertility in the insurance‐covered group may reflect broader and easier access to care. Since patients with tubal pathology often require IVF as a first‐line treatment, reduced financial barriers may have facilitated earlier decision to challenge IVF among these individuals. The insurance‐covered group also included a greater proportion of patients with already one child or history of previous miscarriage. This pattern may indicate the case as follows. Individuals who desire second child were encouraged to pursue ART due to affordable cost by insurance coverage. Other individuals who had become exhausted mentally or financially by miscarriage or multiple treatment failure started again due to easier access to ART.

Multivariable analysis identified several factors associated with the clinical decision‐making process for MBT. Increased maternal age and a higher number of previous failed ETs were associated with a higher likelihood of choosing MBT. Conversely, a history of prior childbirth was associated with a reduced likelihood. Most importantly, health insurance coverage emerged as a primary correlate, with its implementation being associated with a 6.59‐fold increase in the odds of MBT. These findings suggest that the trend toward MBT among older patients may reflect an age‐related decline in fecundity, potentially exacerbated by the finite number of transfer cycles permitted on female ages under the public health insurance policy. In contrast, patients with a history of childbirth were more likely to avoid the risks of MP attributed to MBT. Our investigation into the incidence of MP further underscored the important role of MBT as a key mediator. In the model unadjusted for MBT, insurance coverage, previous ETs, childbirth history, and transfer of high‐quality blastocysts were associated with MP. However, upon adjustment for MBT, the observed associations between insurance coverage, previous ETs, childbirth history, and MP lost statistical significance, suggesting that these trends were largely accounted for by the practice of MBT. Moreover, in this adjusted model, increasing maternal age emerged as a factor associated with decreased odds of MP, revealing an inverse relationship when confounding by transfer strategy is accounted for. While the transfer of high‐quality blastocysts remained an independent risk factor for MP, the practice of MBT itself presented the high‐risk profile, with an aOR of 61.2. Collectively, these findings highlight that the observed trends in the incidence of MP under the insurance policy are primarily associated with shifts in clinical decision‐making regarding MBT. In order to elucidate further the mechanism underlying the increase in MBT and MP observed in blastocyst transfer performed under the insurance policy, we conducted additional subgroup analyses. Specifically, the age‐stratified analysis revealed a significant interaction between age categories and insurance status. Patients aged 40–43 years exhibited a substantially higher likelihood of undergoing MBT compared to their younger counterparts. These data suggest that the structural constraints of the insurance system, specifically the limitation on the number of permitted ET cycles and the age cutoff, may influence clinical decision‐making as patients and clinicians attempt to maximize the probability of pregnancy within a finite window of opportunity, and that these influences are particularly pronounced for older patients. Furthermore, our results indicate a significant upward trend in both MBT and MP rates as patients progressed through their insured transfer cycles. Among patients under 40 years of age, the MBT rate escalated from 4.4% in the first ET cycle to 74.6% by the sixth, the last insured cycle. A similar pattern was observed in the 40–42 age group, where MBT rates reached 72.6% by the third, the last insured cycle. These trends highlight a potential shift toward a tendency to favor MBT as the number of remaining insured ET cycles diminishes.

Recent analyses from the nationwide ART registry in Japan reported that the overall MET rate of more than 600 facilities declined slightly from 15.4% in 2021 to 15.0% in 2022, and the MP rate remained similar at approximately 3% [14]. The apparent discrepancy between these national findings and the results of our study must be interpreted with caution, taking into account differences in the observation period and the types of embryo transfer cycles analyzed. The nationwide analysis evaluated the immediate impact of the introduction of public insurance coverage using data from 2021 and 2022, with the post policy observation window limited to the first 9 months after implementation (April to December 2022). Consequently, it is likely that relatively few patients had reached the maximum limit numbers of insurance covered cycles during that period. Moreover, although the national dataset included both cleavage stage embryos and blastocyst transfers, our study focused exclusively on blastocyst transfer cycles. By excluding transfers of cleavage stage embryos, which generally have lower implantation potential compared to blastocyst and are more likely to be chosen for MET, we might have shown more distinctively the increase in MET‐related clinical decision making of blastocyst transfer. In addition, reports from Japan's ART registry indicate that the number of multiple pregnancies increased sharply in 2023, reaching the highest level in the history of the registry [15]. This surge has drawn an argument regarding the potential contribution of the insurance coverage introduced in 2022.

Multiple previous studies have reported that the transfer of multiple embryos is definitely associated with an increased incidence of multiple gestations [16, 17, 18, 19, 20]. It has further been suggested that ET strategies in ART may be profoundly influenced either by national health policies or financial incentives. In Sweden, a reduction in the number of embryos transferred by guideline has successfully led to a dramatic decline in multiple birth rates while maintaining satisfactory live birth rates, demonstrating that the introduction of single embryo transfer (SET) markedly reduced the incidence of multiple births following ART [21]. Similarly, in Belgium, legislative reform in 2003 linked reimbursement to a new transfer policy aimed at reducing multiple births, resulting in a sharp decline in the multiple birth rate without compromising clinical pregnancy rates [22, 23, 24]. These examples underscore that economic incentives and policy design can exert a decisive influence on ET practices and, consequently, on MP rates. While insurance coverage can alleviate the financial burden on patients, the Japanese national health insurance policy imposes limitations on the number of treatment cycles eligible for coverage depending on the female patient ages. This restriction may encourage the patient to choose MET in order to maximize the probability of pregnancy knowing the risk of multiple gestation. At the same time, there is a desire among patients to minimize the reduction of remaining insured ET cycles. From the perspective of embryo number, when patients have two embryos available, performing SET twice would consume two insured cycles, whereas choosing double embryo transfers would use only one.

MP is well known to be considered substantially higher risks of serious maternal and neonatal complications compared with singleton pregnancies. The prevalence of anemia and iron deficiency is markedly elevated in women with multiple gestations, with significantly higher rates than in singleton pregnancies [25]. Such anemia has been associated with adverse neonatal outcomes, including lower Apgar scores and increased admission rates to neonatal intensive care units [26]. Pregnancies involving more than one fetus are associated with increased risks of preterm delivery, low birth weight, infant disability, and infant mortality [27, 28, 29, 30, 31]. Whereas 53.8% of multiples are born with low birth weight (< 2,500 g), the corresponding figure for singletons is only 3.4%, indicating that multiple births substantially increase the proportion of low‐birth‐weight infants [32]. Multiple gestations also elevate the risk of hypertensive disorders of pregnancy, including preeclampsia, with this trend being particularly pronounced in ART‐conceived multiples [33, 34, 35]. An increased number of embryos transferred may further raise the risk of preeclampsia, whereas SET has the potential not only to reduce multiple births and preterm deliveries but also to lower the risk of preeclampsia [36]. Women with MP have 1.28 times higher odds of developing gestational diabetes compared to those with singleton pregnancies [37]. It has been reported that multiple gestation is an independent risk factor for severe postpartum hemorrhage, with the risk being 3.23 times higher for cesarean deliveries and 6.68 times higher for vaginal deliveries [38, 39, 40]. The presence of multiple gestations also emerged as a potent determinant significantly associated with cesarean section delivery, reflecting the heightened risk of obstetric complications such as malpresentation and necessitating intervention [41].

## 5 Limitations

This study has several limitations. Its retrospective design introduces potential residual confounding despite multivariable adjustment. As a single center analysis, generalizability to other Japanese institutions may be limited. The restriction to blastocyst transfer cycles, excluding cleavage stage and 2 step embryo transfers, may not fully reflect practice patterns across all ART modalities. Moreover, although we identified increased MP rates, long term perinatal and neonatal outcomes were not assessed. Prospective multicenter studies are needed to clarify the broader clinical impact of the policy driven changes.

## 6 Implications and Future Directions

The findings of this study carry important implications for clinical practice and reproductive health policy in Japan. Clinicians should be particularly attentive when counseling patients nearing their insured cycle limits, as increasing MBT use suggests that financial and psychological pressures may lead to underestimation of the risks associated with multiple gestations. From a policy perspective, the current age‐ and cycle‐based restrictions may unintentionally encourage MBT as a strategy to maximize pregnancy outcomes in the limited insured opportunities. Refining the system, such as easing cycle limits or incorporating incentives that favor SET, may better align the policy framework with the goal of achieving healthy singleton births. Finally, validation using nationwide registry data is essential to determine whether these trends are occurring more broadly and to guide evidence‐based adjustments to national ART policy.

## 7 Conclusion

The expansion of public health insurance coverage on infertility treatment, including IUI and ART, from April 2022 appears to be associated with an increased incidence of MBT and a corresponding rise in MP rates. These findings suggest that the current policy's structural constraints, specifically the finite number of insured cycles in each age group, may create paradoxical incentives that prioritize per‐cycle success over maternal and neonatal safety. To align clinical practice with the fundamental goal of achieving healthy singleton births, a strategic refinement of insurance frameworks, combined with comprehensive patient counseling, is essential to promote the wider adoption of SET.

## Ethics Statement

The research protocol for this study was approved by the Institutional Ethics Committee of IVF Osaka Clinic. Given the study's retrospective design, the requirement for written informed consent was waived. Information regarding the purpose and conduct of the study was disclosed to all potential participants, and an opportunity to opt out was provided.

## Conflicts of Interest

The authors declare no conflicts of interest.

## Data Availability

The data that support the findings of this study are available from the corresponding author upon reasonable request.
